# Tularemia on the rise in Switzerland? A one health approach is needed!

**DOI:** 10.1007/s15010-024-02218-9

**Published:** 2024-03-14

**Authors:** Michael Buettcher, Adrian Egli, Sarah Albini, Ekkehardt Altpeter, Anton Labutin, Valeria Guidi, Mauro Tonolla, Reto Lienhard, Onya Opota, Patrizia Schmid, Tsering Wuethrich, Kristina M. Schmidt, the TULAR-CH working group, Peter Keller, Pascal Bittel, Christoph Aebi, Nina Schöbi, Csongor Deak, Christa Relly, Silke Bruhn, Dominik Müller, Geraldine Jost, Sara Schütz

**Affiliations:** 1 Paediatric Infectious Diseases, Department of Paediatrics, Children’s Hospital of Central Switzerland (KidZ), Lucerne Cantonal Hospital, Spitalstrasse, 6000 Lucerne, Switzerland; 2 Faculty of Health Science and Medicine, University Lucerne, Lucerne, Switzerland; 3 Paediatric Pharmacology and Pharmacometrics Research Center, University Children’s Hospital Basel (UKBB), University Basel, Basel, Switzerland; 4 Institute of Medical Microbiology, University of Zurich, Zurich, Switzerland; 5 Coordination Commission of Clinical Microbiology, Swiss Society of Microbiology, Cheseaux-sur-Lausanne, Switzerland; 6 Section for Poultry and Rabbit Diseases, Institute for Food Safety and Hygiene, Vetsuisse Faculty, University of Zurich, Zurich, Switzerland; 7 Federal Office of Public Health, Bern, Switzerland; 8 Institute of Microbiology, University of Applied Sciences of Southern Switzerland–SUPSI, Mendrisio, Switzerland; 9 ADMED Microbiologie, La Chaux-de-Fonds, Switzerland; 10 Spiez Laboratory, Federal Office for Civil Protection (FOCP), Spiez, Switzerland; 11 Swiss National Reference Center for Highly Pathogenic Bacteria (NABA), Spiez, Switzerland; 12 Present Address: Swiss National Reference Center for Tick-Borne Pathogen, CNRT, La Chaux-de-Fonds, Switzerland; 13 Institute of Microbiology, Lausanne University and Lausanne University Hospital, Lausanne, Switzerland; 14 Department of Plant Biology, Microbiology Unit, University of Geneva, Geneva, Switzerland

**Keywords:** Tularemia, Francisella, Switzerland, Zoonosis, Ticks, One Health

## Abstract

In the last 10 years, an increase in tularemia cases has been observed in both humans and animals in Switzerland. In these, infection with *Francisella tularensis*, the causative agent of the zoonotic disease tularemia, can occur through arthropod vectors or contact to infected animals or exposure to contaminated environmental sources. Currently, we are only able to postulate potential aetiologies: (i) behavioral changes of humans with more exposure to endemic habitats of infected arthropod vectors; (ii) an increased rate of tularemia infected ticks; (iii) increasing number and geographical regions of tick biotopes; (iv) increasing and/or more diverse reservoir populations; (v) increasing presence of bacteria in the environment; (vi) raised awareness and increased testing among physicians; (vii) improved laboratory techniques including molecular testing. To approach these questions, a one-health strategy is necessary. A functioning collaboration between public health, human medicine, and diagnostic and veterinary units for the control of tularemia must be established. Furthermore, the public should be included within citizen-supported-science-projects.

## Introduction

Zoonotic diseases, which are transmitted from animals to humans, are responsible for approximately 70% of emerging infectious diseases globally [1]. As climate change and global warming persist, mathematical modeling predict a concomitant increase in the incidence of zoonotic infections, particularly vector-borne diseases [2]. A key pathogen in this context is *Francisella tularensis* (Ft), the causative agent of tularemia. *F. tularensis* is a fastidious, facultative intracellular non-spore-forming, non-motile, Gram-negative coccobacillus. With the ability to infect over 100 species of wild and domestic vertebrates [3] as well as more than 100 invertebrate species [4], *F. tularensis* poses a significant threat to public health. Human infection with *F. tularensis* occurs via various routes, including arthropod bites, injuries caused by infected animals, consumption of contaminated food and water sources, contact with contaminated fomites, and occasionally through the inhalation of aerosols. Due to the bacterium’s stability in the environment, its remarkably low infective dose, and its high virulence when inhaled as aerosol, *F. tularensis* is classified as risk group 3 pathogen and designated as a category A high-priority bioterrorism agent. Worldwide four subspecies of *F. tularensis* are known. In Switzerland, tularemia is caused however, by *F. tularensis subsp. holarctica* (type B). Generally, this subspecies exhibits less virulence than *F. tularensis subsp. tularensis* (type A) which is prevalent in North America.

**Fig. 1:**
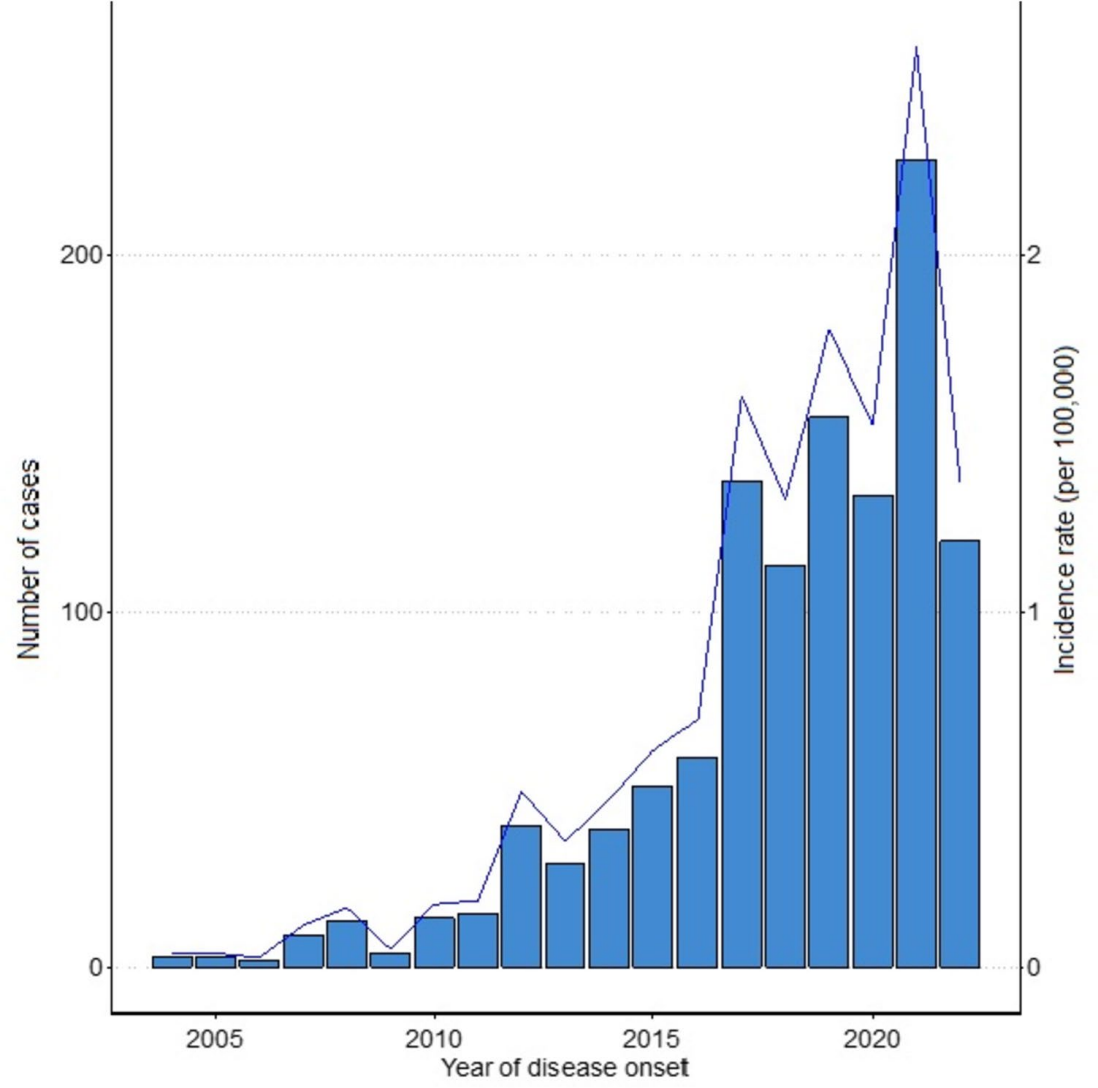
Annual trends in reported human tularemia cases (all age groups) in Switzerland. The figure displays the incidence per 100′000 and number of reported tularemia cases in humans since the implementation of the ordinance mandating the reporting of observations of communicable diseases, including tularemia, in 2004. Reporting is based on laboratory (serology, PCR, culture) and clinical data. Data provided by the Federal Office of Public Health

Since 1995, in Switzerland, the reporting of tularemia cases to the federal office is mandatory for animals and since 2004 for humans. During the period of 2004–2013, 61 human cases were reported, while during 2014–2022, there were 430 cases, an increase by a factor of 7 (Fig. 1) [5]. Neighboring countries such as Austria, but also Sweden and Finland have also documented rising trends in reported human cases [6]. Similarly, the number of animal cases rose from three cases (all hares) in 2011 to 10 cases (one monkey, one cat, eight hares) in 2021 (increase by factor 3.3). The underlying factors driving these trends in Switzerland have yet to be fully elucidated.

As tularemia can affect humans and animals, is transmitted by vectors or via environmental sources, a One Health perspective, including a collaborative and transdisciplinary approach, is necessary. The aim of this brief report is to look at these different aspects and discuss potential research priorities in a One Health perspective.

## Discussion

### Knowledge gaps in the human host

Tularemia manifests with a wide spectrum of clinical presentations depending upon factors, such as the transmission route, the affected entry site, and the subspecies of the pathogen involved. *F. tularensis subsp. tularensis* (type A) is known to cause more severe disease forms compared to *F. tularensis subsp. holarctica* (type B). Ulceroglandular and glandular tularemia are the most commonly observed syndromes in humans, followed by the less common oculoglandular, oropharyngeal, pneumonic, and typhoidal tularemia syndromes. Atypical manifestations are diverse and include fever of unknown origin, endo- and pericarditis, appendicitis, peritonitis, liver abscess, cerebellitis with ataxia, meningitis, encephalitis, osteoarticular infections, rhabdomyolysis, and venous thrombosis [7]. Treatment of tularemia typically involves the use of antibacterial agents including aminoglycosides (gentamycin and streptomycin), fluoroquinolones (ciprofloxacin, levofloxacin, and moxifloxacin) or doxycycline [7]. Despite the current understanding of tularemia, there are several knowledge gaps that need further investigation:

1. Regional (glandular, ulceroglandular, oculoglandular, and oropharyngeal) vs. disseminated disease: In regional tularemia, suppuration is a frequent complication, often necessitating hospitalization and surgical intervention. Identification of risk factors could facilitate timely diagnosis and appropriate antibacterial treatment. Additionally, the role of specific clades and host immunological factors in the development of localized disease requires further exploration. This can be achieved by analyzing clinical data from affected humans and animals, alongside comprehensive microbiological data.
2. Subclinical vs. clinical disease course: The reasons for varying individual susceptibility to tularemia remain unclear. Seroprevalence and genetic studies in human hosts could help elucidate these factors.
3. Disease patterns and treatment response between children and adults: Data on clinical presentation, disease spectrum, management, treatment type, and outcomes, particularly in children, are scarce. Treatment options such as ciprofloxacin or doxycycline are often used but formally are off-label. Systematically assessed pharmacokinetic and safety data are missing. Treatment studies in children are essential. Moreover, it is crucial to determine whether the currently recommended dosages are optimal for both children and adults with tularemia.
4. Serology vs. culture/PCR diagnostics: Prompt and accurate diagnosis is crucial for effective management of tularemia. While serology testing can be informative, pathogen-specific IgG may persist for years and may not accurately represent recent exposure, unless a titer increase is demonstrated. More specific confirmatory tests are often required. In addition to culture-based diagnosis, direct pathogen detection can be achieved through pathogen-specific PCR or bacterial broad-spectrum PCR targeting the 16S rRNA. However, these diagnostic tools are underutilized or not readily available in many settings. There are two PCR-based approaches used by most laboratories in Switzerland: species-specific PCR and 16S + based broad-spectrum PCR, followed by sequencing. Some laboratories use different systems (e.g., different primers). The use of PCR during the recent years is shown in Table 1. Although not giving a fully comprehensive picture, it is a snapshot from the largest laboratories where samples are submitted to, showing a clear increase of PCR requests.

**Table 1:** Diagnostic data provided on the positivity rate of PCR testing on samples in suspected tularemia cases from 2016 to 2022 from the four most consulted laboratories in Switzerland (Spiez, Bern, Zurich, and Lausanne)

Year	2016	2017	2018	2019	2020	2021	2022
Laboratories	2	2	3	3	3	4	4
Samples (total), N	160	194	327	398	388	617	631
Samples (positive), N	20	23	36	37	43	98	57
Positivity rate (%)	13	12	11	9	11	16	9

This is not a complete list of all potential Swiss Tularemia PCR Testing
5. Disease awareness: It is imperative to assess whether healthcare professionals, including pediatricians or adult practitioners in both hospital and out-patient settings, are sufficiently informed about tularemia and its manifestations. Public health initiatives should aim to improve knowledge about the disease and its prevention among healthcare providers and the general population.

### Reservoirs and ecology of *F. tularensis*: knowledge gaps and implications for disease control

Our understanding of the role of reservoir hosts and environmental reservoirs for *F. tularensis* is currently incomplete. Contaminated water and soil play an important environmental reservoir role, as *F. tularensis* is highly adaptable and can persist in these environments for extended periods. Wild lagomorphs (hares and rabbits), small rodents, and arthropods (ticks, flies) serve as reservoirs for *F. tularensis*. However, lagomorphs and small rodents do not represent a long-term reservoir source as they often die from tularemia, particularly subspecies tularemia and less so when infected with subspecies holarctica [8]. This is not true for rats, who often survive infection [9]. In Switzerland, the terrestrial life cycle is common, so it is postulated that ticks, lagomorphs, and rodents are the main reservoir hosts [9, 10]. However, given the scarcity of European wild rabbit (*Oryctolagus cuniculus*) and the vulnerable status of the European brown hare (*Lepus europaeus*) in Switzerland, alternative reservoir species, such as abundant and widespread small rodent species, must be considered [10]. Future studies on *F. tularensis* distribution should engage veterinarians and biologists as experts to identify the most suitable host species for each geographic area and collect the most relevant samples.

### Distribution, vectors, and seasonality

Two studies analyzing data from 2009 to 2015 and 2017 to 2022 identified tick-infested areas primarily north of the Alps as risk regions for human tularemia cases in Switzerland [11, 12]. Consequently, two *F. tularensis* clades were reported from Switzerland: the B.11 genotype, found in Western Europe and Switzerland, and the derived B.45 genotype, spread across most of the Swiss terrain below 1500 m altitude. Although of low prevalence (0.02%), *F. tularensis* was detected in *Ixodes ricinus* tick species in Switzerland [11, 12]. However, other potential arthropod vectors besides ticks have not been investigated for *F. tularensis* presence in Switzerland.

Hence, several unanswered questions concerning ticks and other potential sources should be investigated: (i) the detection limit of *F. tularensis* in ticks and mosquitoes has not been definitively determined. A study introducing a multi-target real-time PCR for *F. tularensis* detection in complex samples suggests sensitivity levels between 1 and 44 genome equivalents, depending on the target gene [13]; (ii) only a few studies have examined *F. tularensis* survival in ticks [14] and mosquitoes [15] under laboratory conditions. Cultivating this fastidious bacterium is challenging and necessitates BSL-3 facilities (in Switzerland this is necessary for all *F. tularensis* subspecies, *including. holarctica*); (iii) other sources of *F. tularensis* infections, such as water or soil, need further evaluation.

Consequently, it is crucial to conduct methodological studies to understand the degree of underrepresentation in the existing data, encompassing more candidate reservoirs and potentially new vectors to better comprehend the environmental dynamics of the *F. tularensis* life cycle. Enhancing our knowledge of the ecological niches of *F. tularensis* in Switzerland could help redefine risk areas and to control case numbers in humans and animals. Since 2017, the Swiss FOPH data show that tularemia cases have been reported earlier each year, suggesting a shift in seasonality toward former colder months [5]. Furthermore, data from Swiss Federal Office of Meteorology and Climatology (SwissMeteo) show that average monthly temperatures for the month of July in northern Switzerland (below 1000 m above sea level) show an upward trend with + 0.5 ℃ per 10 years from 1961 to 2023. The warming trends were also statistically significant across the whole of Switzerland when looked at for each season [16]. Relative humidity of 70–80% close to the soil including adequate vegetation cover is favorable for ticks. These tick favorable areas have spatially increased in northern Switzerland as observed in a study during the years 2009 and 2018 [17]. Multiple factors may contribute to these phenomena, including: (1) better reporting due to heightened awareness among physicians, (2) improved implementation of surveillance systems, (3) climate changes affecting the ecology and distribution of reservoir hosts and vectors, (4) an increase in the prevalence of known *F. tularensis* vectors, or (5) the emergence of new vectors.

### The imperative for a One Health approach in Switzerland and beyond

The evident increase in tularemia cases in Switzerland demands a thorough evaluation and a comprehensive strategy to address this pressing public health concern. Given that *F. tularensis* can be transmitted through multiple routes, it is crucial to adopt a One Health approach that fosters interdisciplinary collaboration among experts in public health, human medicine, vector ecology, diagnostics, and veterinary medicine. This holistic approach will enable a more nuanced understanding of the interaction between the environment, animals, humans, and vectors in the transmission cycle of *F. tularensis*. Modern surveillance should encompass not only traditional epidemiological data, such as case numbers in animals and humans, but also incorporate cutting-edge techniques like whole genome sequencing. This integrated approach will facilitate the monitoring of *F. tularensis* distribution with high spatio-temporal resolution. Platforms such as the Swiss Pathogen Surveillance Platform (www.spsp.ch) could be employed to collect, manage, and analyze data, enhancing surveillance and research efforts.

Furthermore, engaging the public through citizen science projects may be helpful to contribute to this One Health approach, by for, e.g., the collection and submission of data on vector localization, environmental factors, and by the delivery of removed vectors from humans and animals for pathogen detection and monitoring. This collaborative effort will allow for a more comprehensive understanding of the dynamics of tularemia and inform targeted interventions.

The call for action should not be limited to Switzerland; it is imperative to extend this One Health approach across Europe to effectively address the challenges posed by tularemia and other zoonotic diseases. By fostering collaboration among experts from diverse fields, we can work together to ensure the health and well-being of humans, animals, and the environment.

## Data Availability

All data supporting the findings of this report are available within the paper. Epidemiological anonymized data are available from https://www.blv.admin.ch/blv/de/home/tiere/tierseuchen/uebersicht-seuchen/zoonosen.html.
